# Males and females exhibit similar muscle glycogen recovery with varied recovery food sources

**DOI:** 10.1007/s00421-020-04352-2

**Published:** 2020-03-25

**Authors:** Shannon Flynn, Alejandro Rosales, Walter Hailes, Brent Ruby

**Affiliations:** Montana Center for Work Physiology and Exercise Metabolism, College of Integrative Physiology and Athletic Training, The University of Montana, McGill Hall, Missoula, MT 59812 USA

**Keywords:** Glycogen re-synthesis, Post-exercise recovery, Sex differences, Sports supplements

## Abstract

**Purpose:**

Research has elucidated the impact of post-exercise carbohydrate nutrition and environmental conditions on muscle glycogen re-synthesis. However, research has minimally considered the implications of glycogen recovery in females and has mostly focused on commercial sport nutrition products. The purpose of this study was to determine the effects of varied mixed macronutrient feedings on glycogen recovery and subsequent exercise performance in both sexes.

**Methods:**

Males (*n* = 8) and females (*n* = 8) participated in a crossover study. Subjects completed a 90-min cycling glycogen depletion trial, then rested for 4 h. Two carbohydrate feedings (1.6 g kg^−1^) of either sport supplements or potato-based products were delivered at 0 and 2 h post-exercise. Muscle biopsies (glycogen) and blood samples (glucose, insulin) were collected during the recovery. Afterwards, subjects completed a 20 km cycling time trial.

**Results:**

There was no difference between sexes or trials for glycogen recovery rates (male: 7.9 ± 2.7, female: 8.2 ± 2.7, potato-based: 8.0 ± 2.5, sport supplement: 8.1 ± 3.1 mM kg wet wt^−1^ h^−1^, *p* > 0.05). Time trial performance was not different between diets (38.3 ± 4.4 and 37.8 ± 3.9 min for potato and sport supplement, respectively, *p* > 0.05).

**Conclusions:**

These results indicate that food items, such as potato-based products, can be as effective as commercially marketed sports supplements when developing glycogen recovery oriented menus and that absolute carbohydrate dose feedings (g kg^−1^) can be effectively applied to both males and females.

## Introduction

Endurance exercise performance and time to exhaustion have historically been linked to initial levels of glycogen storage within the skeletal muscle (Ahlborg et al. 1967; Bergstrom et al. 1967). Additionally, given the expected depletion rates of glycogen and the finite storage capacity within the muscle, sport nutrition guidelines have developed evidence-based strategies to optimize glycogen recovery after fuel-depleting training or competition. Most commonly, these recommendations suggest a systematic increase in dietary carbohydrate (Blom et al. 1987; Ivy et al. 1988a, b) or mixed diet sources (Ivy et al. 2002; Jentjens et al. 2001; Tarnopolsky et al. 1997; van Loon et al. 2000) in a timely fashion post-glycogen-compromising or -depleting exercise. Collectively, the underlying rates of glycogen re-synthesis are influenced by the extent of glycogen depletion (Price et al. 1994; Zachwieja et al. 1991), the timing of ingestion (Ivy et al. 1988a), dose and amount (Blom et al. 1987; Ivy et al. 1988b; Jentjens et al. 2001; van Loon et al. 2000), the composition as either varied carbohydrate source (Blom et al. 1987; Burke et al. 1993; Ivy et al. 1988a, b; Kions et al. 1990; Reed et al. 1989) or in combination with amino acid blends or protein (Ivy et al. 2002; Jentjens et al. 2001; Tarnopolsky et al. 1997; van Loon et al. 2000; Zawadski et al. 1992), and most recently ambient (Naperalsky et al. 2010) and skeletal muscle temperature (Slivka et al. 2012; Tucker et al. 2012).

When compared to sport nutrition products, food items have demonstrated success in promoting 4-h glycogen re-synthesis rates. Both chocolate milk (Lunn et al. 2012) and fast food items (Cramer et al. 2015) restore glycogen similarly to common commercial sport nutrition products. Chocolate milk (Karp et al. 2006; Lunn et al. 2012; Pritchett et al. 2009; Thomas et al. 2009) and fast food (Cramer et al. 2015) also impact subsequent exercise performance similarly to commercial sport nutrition items. Collectively, these data demonstrate that a variety of dietary strategies can be effective in promoting glycogen re-synthesis following a bout of glycogen-depleting exercise.

Recommendations for optimal re-synthesis appear uniformly applied to both men and women despite a relatively small amount of research including female participants. When comparing women to men, research provides conflicting results regarding both the impacts of glycogen super-compensation (or pre-loading) (James et al. 2001; Paul et al. 2001; Tarnopolsky et al. 2001; Walker et al. 2000) and substrate use during endurance exercise (Devries 2016; Riddell et al. 2003; Roepstorff et al. 2002; Ruby et al. 2002a; Tarnopolsky and Ruby 2001). However, most of the subtle metabolic differences previously demonstrated appear associated with circulating levels of endogenous and exogenous estradiol. This has been the topic of multiple reviews (Tarnoposky and Ruby 2001; Tarnoposky 2008).

These investigations illustrate both similarities and differences between males and females, however, few directly report post-exercise glycogen re-synthesis rates for both sexes (Tarnopolsky et al. 1997). Exogenous provision of adequate substrate in the form of liquid supplements yields comparable glycogen restoration for men and women, but subsequent exercise performance has not been previously evaluated following a post-exercise carbohydrate feeding recovery period (Tarnopolsky et al. 1997).

The purpose of the present investigation was to compare glycogen recovery and subsequent exercise performance in males and females following ingestion of either a potato-derived mixed macronutrient diet or commercial sport nutrition oriented diet. Key dependent measures included rates of muscle glycogen re-synthesis and 20 km cycling time trial performance. We hypothesized that there would be minimal differences in glycogen re-synthesis or exercise performance between the two recovery diets with similar macronutrient distribution. We further hypothesized that males and females would demonstrate similar rates of glycogen re-synthesis when total exogenous carbohydrate was provided proportional to total body weight (1.6 g kg^−1^).

## Methods

### Participants

Recreationally active subjects (*N* = 16; *n* = 8 males and *n* = 8 females) completed the study. The study subjects were healthy, injury-free, and familiar with endurance exercise (refer to Table 2 for descriptive statistics). All of the female subjects were currently using supplemental hormone prescriptions (triphasic norethindrone and ethyl estradiol, oral pill, *n* = 4) or time-release (intrauterine device, *n* = 4) birth control. All subjects completed a Physical Readiness Questionnaire (PAR-Q) and gave written informed consent prior to the study. Prior to subject recruitment and subsequent testing, all procedures were approved by the university’s internal review board (IRB).

### Preliminary testing

Each subject fasted for 4 h and abstained from exercise, alcohol, and caffeine for 24 h prior to an initial visit. After measuring body mass and height, body composition was obtained via hydrodensitometry with corrections for estimated residual lung volume (Goldman and Becklake 1959). Underwater body mass was obtained using an electronic scale (Exertech, Dreshbach, MN, USA) to calculate body density. Sex-specific equations were utilized to convert body density to percent body fat (American College of Sports Medicine ).

Using a cycle ergometer (Velotron, RacerMate Inc., Seattle, WA, USA), peak oxygen uptake (*V*O_2Peak_) and maximal power output (*W*_Max_) were determined for each subject during a graded exercise protocol. The protocol started at 95 W for men and 60 W for women, increasing 35 W every 3 min until volitional fatigue and the achievement of at least two of the following specific max test criteria: respiratory exchange ratio (RER) > 1.10, plateau in *V*O_2_, heart rate (HR) within 10 beats per minute (BPM) of age-predicted max, or rating of perceived exertion (RPE) > 17. During the test, gas exchange was measured using a calibrated metabolic cart (ParvoMedics, Inc., Salt Lake City, UT, USA). *V*O_2peak_ was calculated as the highest 15 s average oxygen uptake and *W*_Max_ was calculated using the following equation: *W*_Max_ = (power output in *W* during last completed stage) + ((final stage time before fatigue in s/180 s) × 35 W).

On the second and third visit, each participant completed a practice (PTT) 20 km time trial (TT) on a computer-simulated course, totaling two PTT. Subjects were instructed to complete the distance as quickly as possible. Subjects were allowed to electronically change speed and shift gears. Distance and time were recorded using RacerMate Inc. software (RacerMate, Inc., Seattle, WA, USA). These sessions were designed to familiarize subjects with the instrumentation, ensuring TT competency prior to the experimental trials.

### Experimental design

**Fig. 1:**
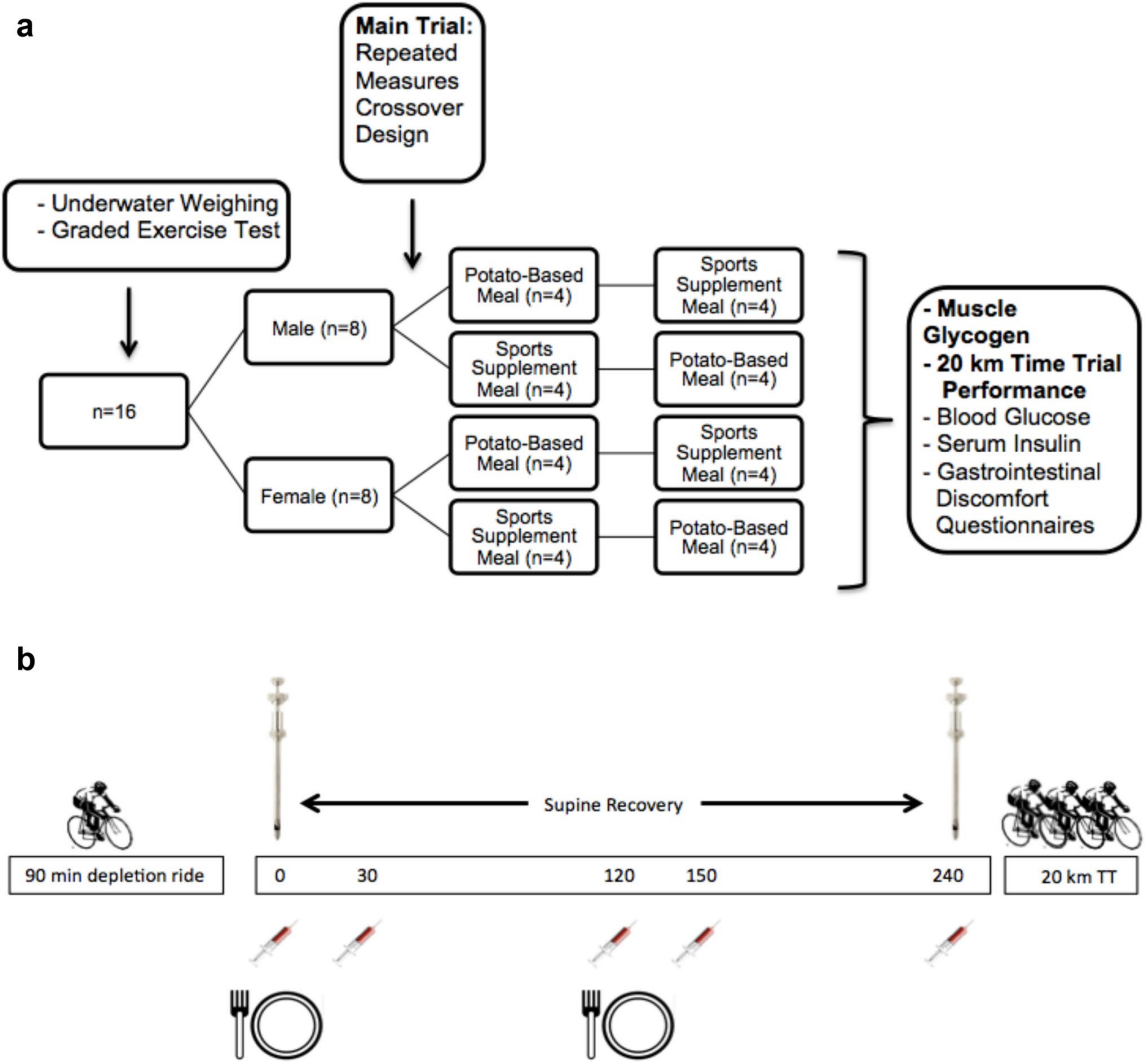
**a** Study design with dependent measures included (primary measures in bold), **b** main experimental trial design

Subjects completed two trials in a crossover, counter-balanced design with 7 days between trials. Females were tested without attempting to control timing across the menstrual cycle. Subjects were instructed to consume a mixed diet the day before each trial and keep a dietary record of all foods and drinks consumed in the 24 h prior to their initial experimental trial. This food record was then used to repeat the same 24 h pre-dietary intake for the subsequent experimental trial. For each laboratory visit, subjects arrived following a 12-h fast, while at the same time refraining from exercise, alcohol, and caffeine for the previous 24 h. For each trial, participants completed a glycogen depletion ride, a 4-h recovery period, and a 20 km TT. During the recovery, subjects consumed one of two mixed macronutrient diets (sports supplement products = SS, potato-based foods = PB, refer to Table 1). To maintain a similar carbohydrate dose for all subjects (approximately 1.6 g kg^−1^), one of four unique serving sizes was assigned to each participant based upon body mass ranges from preliminary testing. Subjects completed gastrointestinal discomfort and meal satisfaction questionnaires and muscle biopsies and blood samples were collected throughout the recovery period. Figure 1 illustrates the experimental design.

**Table 1:** Bolus feeding protocol for 0 h and 2 h post-exercise recovery for body mass of 62–74 kg

	Calories	Fat (g)	Carb (g)	Protein (g)	Sodium (mg)
Sports supplement					
0 h					
Rehydrate Salt Tablet	0	0	0	0	800
Powerade Mountain Berry Blast (591 mL)	130	0	35	0	250
Lara Bar Peanut Butter Chocolate Chip	440	22	52	12	120
Gatorade Prime Energy Chews Fruit Punch (Six Chews)	83	0	20	0	58
Total	653	22	107	12	1228
2 h					
Rehydrate Salt Tablet	0	0	0	0	800
Gatorade Lemon-Lime (591 mL)	140	0	36	0	270
Cliff Mojo Bar Mountain Mix	400	18	46	20	480
Cliff Bloks Energy Chews Mountain Berry (Six Bloks)	117	0	28	0	58.5
Total	657	18	110	20	1609.5
4 h total	1310	40	217	32	2836.5
Potato based					
0 h					
Kraft Pancake Syrup	105	0	28	0	0
Simplot Old European Potato Pancakes	240	10	34	4	620
Mott’s Unsweetened Applesauce	40	0	10	0	0
Great Value Hash Brown	300	16	36	4	540
Total	685	26	108	8	1160
2 h					
Delallo Potato Gnocchi (140 g)	345	0	75	9	840
Prego Traditional Pasta Sauce (120 mL)	70	1.5	13	2	480
Great Value Seasoned Fries (84 g)	175.5	9.4	21	2.3	410
Total	590.5	10.9	109	13.3	1730
4 h total	1275.5	36.9	217	21.3	2890

#### Glycogen depletion protocol during main experimental trials

Subjects completed a 90-min cycle ergometer glycogen-depleting ride. The protocol consisted of a 10-min warm up at 55% *W*_Max_, a series of 10 intervals (2 min at 80% *W*_Max_ followed by 4 min at 50% *W*_Max_) lasting 60 min, 8 min at 60% *W*_Max_, and 12 min at 50% *W*_Max_. Water was consumed ad libitum during the exercise. Exercise occurred in laboratory ambient conditions of 20 °C, 25–30% relative humidity with a light fan. Following the depletion ride, participants rested in a seated or reclined position for a 4-h recovery period. Recovery was completed in the same ambient temperature environment but without the fan.

#### Feeding strategy

At 0 h and 2 h of recovery, participants consumed one of two mixed macronutrient diets with equivalent macronutrient distributions (SS or PB). Nutrition label serving sizes were used to match macronutrients, while items were additionally weighed for accuracy. Each subject consumed 1.6 g carbohydrate kg body weight^−1^ for each feeding. Fat and protein content were also comparable between trials. Table 2 provides details in reference to specific menu items and feeding schedules.

**Table 2:** Descriptive data

Characteristic	Men (*n* = 8)	Women (*n* = 8)	Significance (*p* value)
Age (years)	27.1 ± 6.8	25.1 ± 4.8	NS
Weight (kg)	70.9 ± 8.3	59.2 ± 4.8	*p* < 0.01
Fat-free mass (kg)	62.9 ± 5.7	48.4 ± 4.9	*p* < 0.001
Body fat (%)	14.6 ± 4.2	18.3 ± 5.8	NS
*V*O_2peak_ (L min^−1^)	4.0 ± 0.2	2.8 ± 0.4	*p* < 0.0001
*V*O_2peak_ (mL kg^−1^ min^−1^)	56.7 ± 4.2	46.5 ± 6.6	*p* < 0.01
*V*O_2peak_ (mL kg FFM^−1^ min^−1^)	63.8 ± 5.0	57.2 ± 6.3	NS

Values are means ± SD

*NS* not significant, *p* values by independent *t* test

#### Questionnaires

Subjects completed gastrointestinal discomfort questionnaires at 0, 1, 2, 3, and 4 h of recovery and post-meal questionnaires at 0 and 2 h of recovery. The gastrointestinal discomfort questionnaire gauged hunger, fullness, sickness, and stomach discomfort. The post-meal questionnaire evaluated meal satisfaction, taste, and acceptability. Both questionnaires involved a 150 mm visual analog scale (VAS) with “Not at all” on the left edge and “Extremely” on the right edge of the continuum. Participants placed an “X” along the scale for each question. The distance from “Not at all” in millimeter was divided by 150 mm and reported as a score (Kissileff et al. 2003).

#### 20 km time trial

After the recovery, subjects completed a 20 km cycle ergometer TT described above with the same conditions each trial. Participants were instructed to complete the distance as rapidly as possible. Subjects were not provided encouragement or entertainment and were blinded to time and intensity. Distance and time were recorded similarly to the PTT. Subjects did not consume water during the TTs.

### Tissue and blood sampling and analysis

#### Muscle biopsies

At 0 and 4 h of recovery post-glycogen-depleting exercise, muscle biopsies from the vastus lateralis were extracted using the percutaneous biopsy needle technique and suction (Bergstrom 1975; Evans et al. 1982). Each 4-h biopsy was performed 2 cm proximal to the first location. Each trial utilized a different leg in randomized order. Before the biopsy, 1 mL of 1% lidocaine (without adrenaline) was injected beneath the skin as a local anesthetic. The initial lidocaine numbed a 2 cm^2^ area, wherein another 2–3 mL of lidocaine was injected near the fascia. Following the injection, an approximate 0.5 cm incision was made through the skin and muscle fascia. A Bergstrom biopsy needle was then inserted through the opening into the belly of the vastus lateralis muscle. This practice removed 30 mg of tissue. Excess blood, fat, and connective tissue were subsequently separated from the muscle sample. Remaining tissue was immediately frozen in liquid nitrogen and stored at − 80 °C until muscle glycogen analysis.

#### Blood sampling

Venipuncture technique was used to collect blood samples from an antecubital vein at 0, 30, 120, 150, and 240 min of recovery. Samples were allowed to clot before being spun at 4000 rpm for 15 min in a 4 °C refrigerated centrifuge (Jouan Inc., MR22i). Aliquots of serum were prepared and stored at  − 30 °C until glucose and insulin analyses.

#### Analysis

An enzymatic spectrophotometric method was used to analyze duplicate muscle samples (10–12 mg) for muscle glycogen concentration (Ruby et al. 2005). Following weighing, samples were added to 0.5 mL of 2 N HCl solution. This solution was weighed and incubated for 2 h in a 100 °C oven. Following incubation, the samples were reweighed, reconstituted with distilled water to the original sample weight, and pH normalized using 1.5 mL of 0.67 M NaOH, creating a muscle extract solution. 100 mL of muscle extract solution was added to 1 mL Infinity glucose (HK) liquid stable reagent (ThermoTrace Ltd.). Samples were read at 340 nm on a spectrophotometer, accommodating muscle glycogen concentration calculation using a standard curve. The concentration was expressed as mM kg wet wt^−1^ h^−1^ of muscle.

Blood samples were similarly analyzed for glucose in triplicate with Infinity glucose (HK) liquid stable reagent (ThermoTrace Ltd.) and read at 340 nm on a spectrophotometer. A standard curve was used to calculate blood glucose concentration. An enzymatic spectrophotometric ELISA method was used to analyze insulin from blood samples in duplicate (EIA-2935, DRG International). Mean intra-assay coefficients of variation for muscle samples, glucose, and insulin using these techniques were less than 5%.

### Statistical analysis

Sex-specific demographics were analyzed via two-tailed independent *t* tests (Microsoft Excel, Microsoft Corp., Redmond, WA, USA). Multiple two-way ANOVAs (sex × trial order and sex × trial) with repeated measures were used to analyze PTT and TT performance times (SPSS Inc., Chicago, IL, USA). Muscle glycogen recovery rates were compared using a two-way ANOVA (sex × trial) with repeated measures. A three-way ANOVA (sex × trial × time) with repeated measures was used to examine differences in muscle glycogen, blood glucose, and serum insulin levels between males and females (SPSS Inc., Chicago, IL, USA). Gastrointestinal discomfort and meal satisfaction were compared using a three-way ANOVA (sex × trial × time) with repeated measures. Statistical significance for all analyses was set at a probability of type I error less than 5% (*p* < 0.05). Data in text and tables are expressed as mean ± SD. Graphical data are expressed as mean ± SEM.

## Results

### Subject descriptive data

Sixteen subjects completed the study (eight males, eight females). Descriptive data are presented in Table 2. There was a significant difference between men and women for weight, fat-free mass (FFM), and *V*O_2peak_ (absolute and relative). However, there was no difference between males and females for age, body fat percentage, or VO_2peak_ when normalized for FFM.

#### Blood glucose

**Fig. 2:**
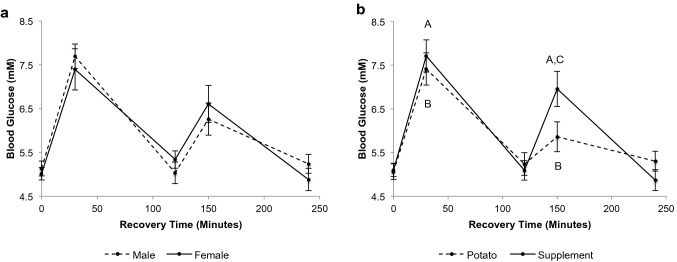
Blood glucose concentration during recovery. **a** Between sexes (*n* = 8 males, *n* = 8 females). **b** Time × trial interaction (*p* < 0.05, *n* = 16 potato, *n* = 16 supplement). **a***p* < 0.01 from 0-min recovery (supplement), **b***p* < 0.0001 from 0-min recovery (potato), **c***p* < 0.01 supplement from potato. Values are mean ± SEM

There was a time × trial interaction for blood glucose (Fig. 2, *p* < 0.05). Blood glucose was elevated at 30 and 150 min (30 min post each feeding) for both PB and SS trials (*p* < 0.0001 and *p* < 0.01 respectively). At 150 min, blood glucose was significantly higher during the SS trial than the PB trial (*p* < 0.01). There was no sex difference in blood glucose at 0, 30, 120, 150, or 240 min post-exercise (*p* > 0.05) and no difference in blood glucose between the diets at 0, 30, 120, or 240 min post-exercise (*p* > 0.05).

#### Serum insulin

**Fig. 3:**
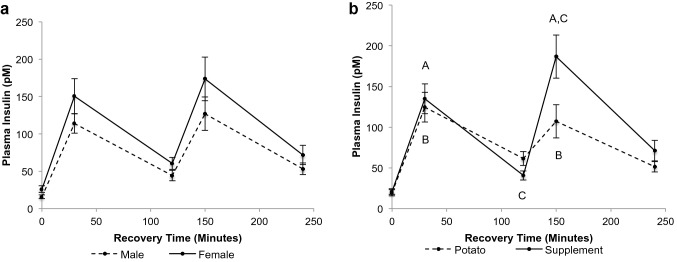
Plasma insulin concentration during recovery. **a** Between sexes (*n* = 8 males, *n* = 8 females). **b** Time × trial interaction (*p* < 0.0001, *n* = 16 potato, *n* = 16 supplement). **a***p* < 0.05 from 0-min recovery (supplement), **b***p* < 0.05 from 0-min recovery (potato), **c***p* < 0.05 supplement from potato. Values are mean ± SEM

There was a time × trial interaction for serum insulin (Fig. 3, *p* < 0.0001). Insulin was elevated at 30 and 150 min post-meal consumption for both PB and SS trials (*p* < 0.05). The PB treatment demonstrated higher insulin at 120 min (*p* < 0.05), while the SS treatment noted higher insulin at 150 min (*p* < 0.05). There was no sexm difference in serum insulin at 0, 30, 120, 150, or 240 min post-exercise (*p* > 0.05) and no difference in insulin between the diets at 0, 30, or 240 min post-exercise (*p* > 0.05).

#### Muscle glycogen

**Fig. 4:**
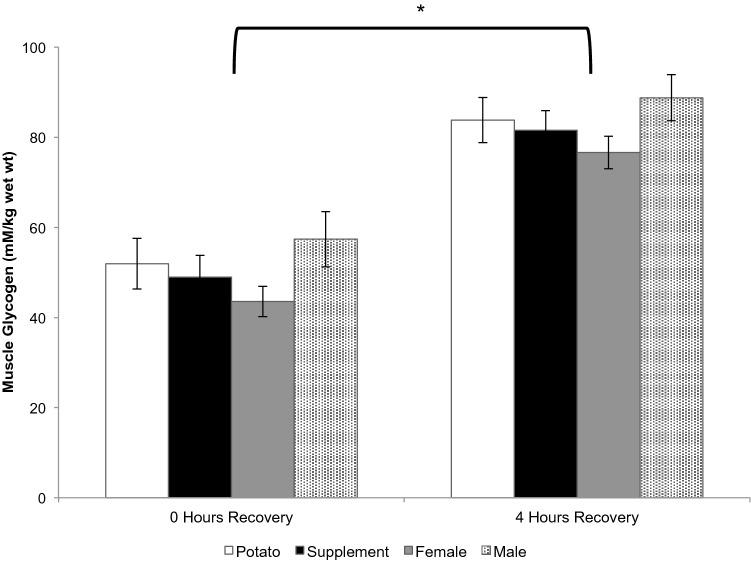
Muscle glycogen concentration during recovery. *p < 0.0001 main effect for time from 0 h recovery. Values are mean ± SEM

There was a main effect for time, where muscle glycogen content increased over the recovery period (Fig. 4, *p* < 0.0001). There was no difference in muscle glycogen content between males and females at 0 h or 4 h of recovery (*p* > 0.05). Correspondingly, there was no difference between males and females in the rate of glycogen re-synthesis (male: 7.9 ± 2.7, female: 8.2 ± 2.7 mM kg wet wt^−1^ h^−1^, *p* > 0.05). SS and PB displayed no differences in muscle glycogen content at 0 h or 4 h of recovery (*p* > 0.05) and no difference in the rate of muscle glycogen re-synthesis (PB: 8.0 ± 2.5, SS: 8.1 ± 3.1 mM kg wet wt^−1^ h^−1^, *p* > 0.05).

#### Questionnaire

For hunger, there was a main effect for time (*p* < 0.0001) and sex (*p* < 0.05). Participants rated hunger higher at 0 h and 2 h of recovery, which was before each meal (0 h: 0.62 ± 0.05, 1 h: 0.45 ± 0.04, 2 h: 0.55 ± 0.04, 3 h: 0.32 ± 0.04, 4 h: 0.31 ± 0.04 VAS scores). Males rated hunger higher than females across trials (male: 0.53 ± 0.05, female: 0.36 ± 0.05). There was a trial × sex interaction for stomach discomfort (*p* < 0.05). Males had less stomach discomfort during the potato trial than the supplement trial (PB: 0.12 ± 0.04, SS: 0.20 ± 0.06). In reference to meal satisfaction, there was a main effect for trial regarding how tasty (*p* < 0.0001), how satisfying (*p* < 0.01), and how acceptable (*p* < 0.05) the meals were. The PB were rated more tasty (PB: 0.65 ± 0.04, SS: 0.43 ± 0.04), satisfying (PB: 0.65 ± 0.05, SS: 0.45 ± 0.05), and more acceptable (PB: 0.59 ± 0.04, SS: 0.47 ± 0.05) than the SS.

#### 20 *km time trial*

There was no interaction or main effect for trial when TT performance was analyzed for order (trial 1: 37.9 ± 3.8, trial 2: 37.6 ± 4.1, trial 3: 38.2 ± 3.8, trial 4: 37.9 ± 4.4 min, *p* > 0.05) or practice and experimental trials (PTT1: 37.9 ± 3.8, PTT2: 37.6 ± 4.1, PBTT: 38.3 ± 4.4, SSTT: 37.8 ± 3.9 min, *p* > 0.05). This indicates there was no learning effect and no difference between SS and PB. However, there was a main effect for sex, with males completing the time trials significantly faster than females (male: 34.7 ± 1.7, female: 41.0 ± 3.0 min, *p* < 0.0001).

## Discussion

Current recommendations for refueling after exercise can be misleading, in particular for recreational athletes who are targeted by commercial marketing (Heneghan et al. 2012; Zytnick et al. 2015). In the current study, males and females resynthesized muscle glycogen at similar rates following glycogen depletion and consumption of two different diets (potato-based food items and common sport supplement products), leading to minimal differences during subsequent exercise performance. When coupled with prior data using fast food items (Cramer et al. 2015), these recent data demonstrate that the exercise recovery carbohydrate oriented feeding recommendations given by health care providers, coaches and trainers can be simplified. Sport and tactical athletes of both sexes can choose a variety of desirable and/or available carbohydrate sources as recovery foods/beverages. Moreover, the diversification of dietary strategies may enhance carbohydrate compliance and sustainability of energy intake.

Similarities between males and females corroborate the findings of Tarnopolsky et al. (1997) who observed comparable rates of post-exercise glycogen re-synthesis in similarly trained males and females (25.5 and 23.5 mM kg dry wt^−1^ h^−1^, respectively) when provided with a mixed macronutrient liquid feeding. Sex similarities in glycogen re-synthesis contradict suggestions that women require unique recovery feeding recommendations when compared to men (Black et al. 2019; Rehrer et al. 2017), indicating that consumers and athletes should be wary of sex-specific marketing related to carbohydrate-rich recovery products. Like Tarnopolsky et al. (1997), the current study found no significant difference between men and women in *V*O_2peak_ when normalized for FFM. However, fitness in our study was lower for both sexes, underscoring the application of recovery recommendations across varied training statuses. A broadened application may be particularly important when considering recovery products that are often marketed to recreational athletes (Heneghan et al. 2012; Zytnick et al. 2015).

Eating before and/or during exercise would be typical of most extended training sessions and competitions for sports athletes and of most workplace scenarios for tactical athletes, while many laboratory investigations use a fasted state. Some research indicates that substrate use sex differences are eliminated when subjects exercise in a fed as opposed to fasted state (Harger-Domitrovich et al. 2007). The current study’s sex similarity also aligns with findings that when men and women are matched for FFM and fitness, whole-body substrate use is similar during exercise (Roepstorff et al. 2002; Ruby et al. 2002a) and that females can super-compensate (or pre-load) their glycogen stores as effectively as males (James et al. 2001; Paul et al. 2001; Tarnopolsky and Ruby 2001; Walker et al. 2000). All of these results are logical when considering that GLUT-4, hexokinase, and glycogen synthase levels and activities are similar between men and women (Lundsgaard and Kiens 2014), which would contribute to limited differences in muscle glucose delivery and storage across sex. These established cellular level comparisons support the outward muscle recovery and subsequent exercise performance responses in the present group of males and females.

Since all of the females were using oral or IUD-derived exogenous hormones for birth control, it is difficult to consider a particular phase of the menstrual cycle. Indeed, past work has sought to control testing relative to the menstrual cycle for measures of glucose kinetics (Zderic et al. 2001; Ruby et al. 2002a, b) and glycogen use (Harger-Domitrovich et al. 2007). The effects of endogenous and/or exogenous estradiol has been demonstrated to reduce the rate of glucose appearance (Ra) and disappearance (Rd) during tightly controlled exercise trials under fasted conditions (Zderic et al. 2001; Ruby et al. 2002a, b; Ruby et al. 1997) but without altering total carbohydrate oxidation. Moreover, the data of Tarnopolsky et al. (2006) demonstrate minimal differences in post-exercise muscle glycogen values between the phases of the menstrual cycle. Despite the potential for hormone (specifically estradiol)-derived variations in substrate oxidation during exercise, it is unclear if the varied hormonal milieu may exert measurable effects on post-exercise carbohydrate delivery and deposition.

The lack of significant difference between PB and SS feedings for rates of glycogen re-synthesis or exercise performance demonstrates that diverse diets can similarly enhance glycogen recovery. The similarity in recovery despite varied carbohydrates extends upon a body of research supporting the use of common foods as an alternate or additional form of sport nutrition as compared to commercial products specifically marketed to improve recovery. Cramer et al. (2015) showed that fast food menu items stimulated glycogen recovery and contributed to subsequent exercise performance as effectively as commercial sports supplement products. Other research has demonstrated that golden raisins and chocolate milk are as effective as sports supplements for providing carbohydrate during (Rietschier et al. 2011) and after (Karp et al. 2006; Lunn et al. 2012; Pritchett et al. 2009; Thomas et al. 2009) exercise. While all of these investigations included a measure of performance, only Rietschier et al. (2011) measured blood glucose during exercise and Lunn et al. (2012) measured muscle glycogen during recovery. Importantly, during the current study, the PB diet was rated higher for taste and satisfaction than the SS and the PB led to lower feelings of sickness and stomach discomfort for males. The positive questionnaire ratings for PB as compared to SS indicate that it could be easier and more agreeable for athletes to consider commonplace foods rather than exclusively consume commercial products marketed as sport-specific recovery products. Evidence shows that many athletes, especially females, have a lower overall energy intake and consume less than the recommended amounts of carbohydrate (Baranauskas et al. 2015; Black et al. 2019; Burke et al. 2001; Masson and Lamarche 2016; Shriver et al. 2013). As a possible solution, dietary diversity tends to increase total caloric consumption (de Oliveira et al. 2018). If athletes are encouraged to consume a variety of foods they enjoy, the likelihood of carbohydrate compliance and sustainable dietary design may be enhanced. Simple recommendations may be particularly critical during situations where total daily energy expenditure meets or exceeds 3 × BMR, such as for occupational athletes like wildland firefighters (Ruby et al. 2002b) or tactical athletes like Marines in combat scenarios (Hoyt et al. 1991).

The subtle differences in glucose and insulin in the current study did not impact the overall rate of glycogen re-synthesis, showing that possible variances in carbohydrate digestion and absorption had limited effect on the delivery of carbohydrate to the muscle. Similar to other studies using a variety of feeding strategies (Ivy et al. 1988a, b, 2002), blood glucose and insulin rose quickly following the 0 and 2-h feedings before returning to baseline. While there was no difference in response between PB and SS at 30 min after the 0-h feeding, there was a difference at 30 min after the 2-h feeding (150 min), with PB eliciting a significantly lower glucose and insulin response compared to SS. The different blood responses could result from differences in the factors impacting gastric emptying between the SS and PB for the 2-h feeding. Although gastric emptying was not measured in the current study, previous research shows that faster emptying can lead to quicker glucose absorption in the small intestine (Macdonald 1996). Other investigations indicate that gastric emptying is enhanced with decreased fat (Frost et al. 2003), fiber (Vincent et al. 1995), and increased liquids (Hellström et al. 2006). Although the SS and PB at both feedings were matched for fat and protein content, both PB had higher fiber content, while both SS had a higher portion of calories from liquid sources. The lack of difference in the glucose and insulin response following the 0-h feeding could indicate that gastric emptying has varying levels of influence based on timing. For example, directly following exercise, muscle glycogen is most depleted so gastric emptying may have less impact than after the 2-h feeding when glycogen re-synthesis has already commenced. Alternately, the different blood responses might be caused by differences in the content of simple sugars between the SS and PB for the 2-h feeding. While the 0-h feedings had a similar proportion of carbohydrate calories from simple sugars, the SS meal at 2 h had a higher simple sugar content, potentially explaining our results. Regardless of the cause of subtle blood differences, the rates of overall muscle glycogen recovery were minimally affected.

The current study does not show evidence suggesting that females should be fed differently than males in regards to post-exercise carbohydrates outside of adjusting total carbohydrate content relative to total body mass (g CHO per kg body mass). Nonetheless, there are some limitations. The investigation did not consider protein, fat, micronutrient, or specific amino acid needs that may differ between sexes. However, West et al. (2012) demonstrated minimal differences between males and females in myofibrillar protein synthesis (MPS) after resistance exercise when subjects were provided with 25 g of whey protein post-exercise. Moreover, the similarities in MPS between males and females occurred despite significantly higher testosterone in males. Although further research could consider whether recommendations for other nutrients should differ between males and females, it is important to link suggested recommendations to outward measures of exercise performance versus theoretical biomarkers of performance. It is also important to note that exogenous carbohydrate during exercise may alter endogenous depletion of glycogen (De Bock 2005) and rates of subsequent glycogen re-synthesis (Reinert et al. 2009). Our study utilized a fasted state, but athletes often work, train, and compete in a fed state, so additional research using alternate feeding strategies may be warranted. Finally, although we did not modify environmental conditions during the current study, consideration of other factors during recovery is critical. Recovering in room temperature ambient conditions (Naperalsky et al. 2010; Slivka et al. 2013) and with warmed muscles (Slivka et al. 2012; Tucker et al. 2012) will enhance glycogen re-synthesis as demonstrated by prior research on males. Environmental elements have not yet been investigated in women.

## Conclusion

The findings of the current study regarding muscle glycogen re-synthesis and exercise performance can be applied to both male and female sport and tactical or occupational athletes of various fitness levels who require glycogen restoration following extended periods of work or between multiple bouts of work in a single day. As long as an adequate amount of carbohydrate (1.6 g kg^−1^) is provided at multiple intervals during recovery, the source of macronutrients for men and women may be diverse and need not be limited to exclusively commercial sport nutrition products. Variation in recovery food sources may enhance carbohydrate compliance, contributing to sustainable recovery dietary design. The uniformity and flexibility of these recommendations simplify and potentially decrease the cost of recovery feeding plans, a notion that may be particularly important for recreational athletes who may be susceptible to commercial marketing.

## Abbreviations

ANOVA: Analysis of variance
BBM: Beats per minute
CHO: Carbohydrate
FFM: Fat-free mass
HR: Heart rate
PB: Potato-based foods
PTT: Practice time trial
RER: Respiratory exchange ratio
RPE: Rating of perceived exertion
TT: Time trial
SS: Sport supplement products
VAS: Visual analog scale
*V*O_2Peak_: Peak oxygen uptake
*W*_Max_: Maximal power output
